# Human Monoclonal Antibodies as a New Class of Antiinfective Compounds

**DOI:** 10.1155/2013/297120

**Published:** 2013-09-11

**Authors:** Roberto Burioni, Alois B. Lang, J. Donald Capra

**Affiliations:** ^1^Medical School, Università Vita-Salute San Raffaele, 20132 Milan, Italy; ^2^GeNeuro SA, 1228 Geneva, Switzerland; ^3^Oklahoma Medical Research Foundation, Oklahoma City, OK 73104, USA

The concept of using “magic bullets” in the fight against infectious diseases was originally proposed by Paul Ehrlich, one of the founding fathers of immunology and of the basis of antiinfective therapy [1]. These “magic” compounds should have been able to target microbes without harming the infected host. Actually, this concept is the mainstay of antiinfective therapy, as we still know it today. What Dr. Ehrlich did not and could not foresee was the microbial ability of using “magic tricks” against therapeutic compounds, that is, of developing resistance mechanisms. This is true for all groups of pathogens and all classes of antiinfective drugs, making the need of reliable alternatives everyday more compelling [2–5]. 

Since their first description in 1975 [6], monoclonal antibodies (mAbs) have been depicted as ideal “magic bullets” due to their extremely specific mode of action, associated with an extreme biotechnological versatility [7]. A mAb with potential therapeutic utility should fulfill at least the following three conditions: (i) specific binding to the molecular target by the antigen-binding fragment (Fab) domain, (ii) effective, but controlled, effector functions activated by binding of the constant crystallizable fragment (Fc) region to specific receptors of immune cells, and (iii) good pharmacokinetic characteristics. The first mAbs were exclusively of animal (murine) origin, with dramatic potential drawbacks in terms of high immunogenicity, short half-life, and low capacity of activating Fc-mediated effector functions when administered to patients. These potential and sometimes actual problems were addressed by engineering the constant regions of an antibody molecule leading first to chimeric (originally murine mAbs with a human Fc fragment) and then to humanized (all human mAbs, only keeping the *complementarity determining regions* (CDRs) of the original mouse mAbs) antibodies [8]. In the last two decades, novel techniques allowed the possibility of dissecting directly the human “antibodyome,” allowing the selection of fully human mAbs [8]. Different approaches aimed at the regulation of the Fc-mediated effector functions have also been described [9]. Several of these “novel” mAbs are finding or will find their way into the clinics in the next few years [9]. 

However, almost all of the licensed therapeutic mAbs are directed against nonmicrobial antigens and are used in autoimmune or neoplastic diseases [9]. Several factors may have contributed to such a “minority report” in the use of mAbs as anti-infectious agents, such as the availability of effective drugs or prophylactic strategies, the extreme variability and complexity of most of the surface-expressed microbial antigens, especially in more evolved microbial pathogens, such as bacteria, fungi, and parasites [10, 11]. 

In this special issue of Clinical and Developmental Immunology, all these aspects are covered by six review articles and four research articles discussing the possible use of human and humanized mAbs against bacterial, viral, and fungal diseases. The different phases of the development of a mAb are discussed, starting from the identification of a potentially effective microbial target and the choice of the potentially most fruitful biotechnological strategy, to importantly the final characterization of each selected mAb. As an example, in the paper by M. Castelli et al., the use of bioinformatic tools in the definition of a mAb epitope is widely discussed, evidencing their possible important application in the novel field of epitope-based vaccinology [12, 13]. The use of mAbs endowed with different biological activities in the study of novel approaches in the investigation of clinically important “emerging” pathogens is also considered, as in the review article by R. A. Diotti et al. intriguingly proposing a novel mAb-based perspective in the study of JCV-associated progressive multifocal leukoencephalopathy [14].

We are certain that the readers of this special issue will find several interesting points of discussion in the published papers, even if not working on the specific microbiological topics discussed. They will certainly agree with us that it is time to rediscover Ehrlich's “magic” in the use of mAbs as anti-infectious agents and that some effective novel anti-infectious “bullets” may find their way into the clinics very soon.

*Roberto Burioni* *Alois B. Lang* *J. Donald Capra*

## References

[1] Ehrlich P (1878). *Beiträge zur Theorie und Praxis der histologischen Färbung*.

[2] Wyles DL (2013). Antiviral resistance and the future landscape of hepatitis C virus infection therapy. *The Journal of Infectious Diseases*.

[3] Gupta N, Limbago BM, Patel JB, Kallen AJ (2011). Carbapenem-resistant enterobacteriaceae: epidemiology and prevention. *Clinical Infectious Diseases*.

[4] Butler MS, Cooper MA (2012). Screening strategies to identify new antibiotics. *Current Drug Targets*.

[5] Finley RL, Collignon P, Larsson DG (2013). The scourge of antibiotic resistance: the important role of the environment. *Clinical Infectious Diseases*.

[6] Kohler G, Milstein C (1975). Continuous cultures of fused cells secreting antibody of predefined specificity. *Nature*.

[7] Teillaud JL (2012). From whole monoclonal antibodies to single domain antibodies: think small. *Methods in Molecular Biology*.

[8] Clementi N, Mancini N, Solforosi L, Castelli M, Clementi M, Burioni R (2012). Phage display-based strategies for cloning and optimization of monoclonal antibodies directed against human pathogens. *International Journal of Molecular Sciences*.

[9] Jiang X-R, Song A, Bergelson S (2011). Advances in the assessment and control of the effector functions of therapeutic antibodies. *Nature Reviews Drug Discovery*.

[10] Mancini N, Solforosi L, Clementi N, De Marco D, Clementi M, Burioni R (2011). A potential role for monoclonal antibodies in prophylactic and therapeutic treatment of influenza. *Antiviral Research*.

[11] Burioni R, Perotti M, Mancini N, Clementi M (2008). Perspectives for the utilization of neutralizing human monoclonal antibodies as anti-HCV drugs. *Journal of Hepatology*.

[12] Clementi N, Criscuolo E, Castelli M, Mancini N, Clementi M, Burioni R (2012). Influenza B-cells protective epitope characterization: a passkey for the rational design of new broad-range anti-influenza vaccines. *Viruses*.

[13] Clementi N, Mancini N, Castelli M, Clementi M, Burioni R (2013). Characterization of epitopes recognized by monoclonal antibodies: experimental approaches supported by freely accessible bioinformatic tools. *Drug Discovery Today*.

[14] Mancini N, Clementi M, Burioni R (2012). Natalizumab-associated progressive multifocal leukoencephalopathy. *The New England Journal of Medicine*.

